# Evaluation of thoracic inlet view plain radiograph in the management of patients with goitre

**DOI:** 10.4314/ahs.v23i4.38

**Published:** 2023-12

**Authors:** Adefemi Oladiran Afolabi, Constantine Ezeme

**Affiliations:** 1 Department of Surgery, College of Medicine, University of Ibadan, Nigeria; 2 Department of Surgery, University College Hospital, Ibadan, Nigeria

**Keywords:** Goitre, retrosternal extension, retrosternal goitre, thoracic inlet, thyroidectomy

## Abstract

**Background:**

Thoracic inlet view radiograph is an investigation for assessing patients with goitre in many centres in the sub-Saharan-region. However, there is paucity of information on its usefulness in the diagnosis of retrosternal goitre (RSG) and in planning for thyroidectomy.

**Method:**

A review of patients with goitre managed in the Division of Endocrine Surgery, University College Hospital, Ibadan, Nigeria, between 2002 and 2014 was done. Data were obtained from Operating Theatre Log and electronic data archive of the Division. Clinical RSG (CRSG) was taken as a gland that the examining fingers could not get below its lower margin and Radiological RSG (RRSG) on thoracic inlet view was any extension of the thyroid gland beyond the thoracic inlet. Intra-operatively, if any part of the gland extends beyond the thoracic inlet it was considered as an RSG.

**Results:**

221 (96.5%) of the 229 patients who had thoracic inlet plain radiograph were included in this study. The Male to Female ratio was 1:5.5. WHO grade III goitre was seen in 56.1% of the patients and 43.9% had grade II goitre. The CRSG, RRSG and Intra-operative RSG were seen in 7.7%, 16.7% and 17.6% respectively. The specificity and sensitivity of clinical examination in determining RSG was 88.7% and 94.1% and that of Thoracic inlet radiograph was 97.8% and 94.6% respectively.

**Conclusion:**

It is a useful study for screening patients with goitre for retrosternal extension, however it could not be used to determine the need for extra-cervical surgical access during thyroidectomy.

## Introduction

The evaluation of patients with goitre involves clinical assessment, biochemical assay, fine needle aspiration cytology and imaging studies. The imaging method employed depends on the clinical presentation, availability, and affordability.

The thoracic inlet view radiograph is one of the imaging modalities used for the evaluation of patients with goitre for retrosternal extension, which is usually seen as a soft tissue opacity in the retrosternal area. The images are acquired in the antero-posterior and lateral projections.^1^ The films could show the extension of the goitre beyond the sternal notch but not its attachment to intrathoracic structures. 2 computed tomography [CT] scan and magnetic resonance imaging [MRI] are the choice investigations for pre-operative assessment of patients with retrosternal goitre for the need of extra-cervical surgical access during surgery. 3 However, in middle and low-income countries of the sub-Saharan Africa, plain radiograph of the thoracic inlet has remained a routine radiological investigation in screening for retrosternal extension of goitre, owing to limited availability and cost of CT scan and MRI.

The thoracic inlet is a kidney-shaped aperture that connects the root of the neck to the thorax. It lies in an oblique transverse plane tilted antero-inferiorly to postero-superiorly, approximately 10cm in the transverse dimension and 5cm in the antero-posterior dimension in adults. The boundaries are T1 vertebral body and costovertebral joints posteriorly, first ribs and the costal cartilages laterally and the superior border of the manubrium anteriorly. It contains neurovascular bundles and structures traversing the neck and thorax. 4

The prevalence of goitre varies from region to region, globally it is estimated to be about 15.8% of the population but 28.3% in Africa. The incidence of retrosternal goitre varies widely and ranges from less than 1% 5 to 26.4%, 6 partly due to the lack of consensus in the definition of retrosternal goitre. Some of the varying definitions in literature includes; when any part of a goitre extends below the thoracic inlet, 7 a goitre extending 3 cm below the sternal notch, 8,9 a goitre extending to the aortic arch 5 and a goitre with more than 50% of the total bulk residing below the thoracic inlet. 10 In thyroidectomies for retrosternal goitre, adequate surgical access may be achieved through cervical incision alone in about 84% but additional access in the form of manubriotomy (3.1%), median sternotomy (6.6%) or thoracotomy (4%) may be required. 11

This study examined the usefulness of thoracic inlet view plain radiograph in preoperative assessment of patients with goitre and in planning for surgery.

## Methods

We conducted a retrospective review of all patients with goitre managed in the division of Endocrine Surgery of the University College Hospital, Ibadan, Nigeria, over a period of 12 years between 2002 and 2014. Data were obtained from an electronic data archive domiciled in the division and operating theatre Log. All the patients who had preoperative thoracic inlet radiograph before thyroidectomy were included. We defined retrosternal extension as any part of the thyroid gland extending beyond the thoracic inlet. 7 The clinical presentation, preoperative radiological investigations, the operative approach (cervical or cervico-thoracic) and operative findings were extracted from the data sources. Clinically retrosternal goitre (CRSG) was taken as a gland that the examining fingers could not get below its lower margin and radiologically retrosternal goitre (RRSG) was defined as any extension of the thyroid gland below the thoracic inlet on thoracic inlet view radiograph. We relied on the reports of the thoracic inlet radiograph by the radiologist in the institution. Intra-operatively, if the lower pole of the gland extends beyond the thoracic inlet in an extended neck it was considered as a retrosternal goitre. The data were analysed using the statistical package for social sciences version 25. Descriptive statistics were summarised in tables, the sensitivity and specificity, and the receiver operating characteristic (ROC) curve was performed.

## Results

Two hundred and twenty-one (96.5%) of the 229 patients with goitre seen over the study period were included in this study. The patients' age range was 13 – 71 years with a mean of 40.7±12.4 years, and a male-to-female ratio of 1:5.5. Simple multinodular goitre was the commonest diagnosis and represents 63.3% of the cases and the proportion of malignant goitre was 6.8%. Anterior neck mass was a constant presenting symptom and 56.1% had WHO grade III goitre. Table 1.

**Table 1:** Patients characteristics and diagnosis

Variable	Frequency (%)
**Age (years); Mean±SD**	40.7±12.4
**Gender**	
Male	34 (15.4)
Female	187 (84.6)
**Clinical diagnosis**	
Simple multinodular goitre	140 (63.3)
Toxic multinodular goitre	20 (9.1)
Toxic diffuse goitre	14 (6.3)
Solitary thyroid nodule	31 (14.0)
Malignant goitre	15 (6.8)
Simple thyroid cyst	1 (0.5)
**WHO grading**	
II	97 (43.9)
III	124 (56.1)

Seventeen patients (7.7%) were found to have RSG on physical examination. This number increased to 37 (16.7%) on evaluation with thoracic inlet plain radiograph and Intraoperatively, RSG was a finding in 39 (17.6%) of the patients.

Total thyroidectomy is the preferred surgery by most general surgeons in the institution and this was performed in 88.7% of the patients as shown in Table 2. Two (5.4%) of the 37 patients with findings of RSG on thoracic inlet radiograph had cervical incision and manubriotomy (cervico-thoracic surgical access) for thyroidectomy, the remaining 35 (94.6%) had only cervical surgical access.

**Table 2:** Distribution of retrosternal extension, surgical access and surgery performed

Variable	Frequency (%)
**Clinical RSG**	
Yes	17 (7.7)
No	204 (92.3)
**Radiological RSG**	
Yes	37 (16.7)
No	184 (83.3)
**Intraoperative RSG**	
Yes	39 (17.6)
No	182 (82.4)
**Surgical Access for radiological RSG**	
Cervical incision	35 (94.6)
Cervical incision + Manubriotomy	2 (5.4)
**Type of surgical Operation**	
Total thyroidectomy	195 (88.7)
Subtotal thyroidectomy	12 (5.4)
Near Total thyroidectomy	8 (3.6)
Completion thyroidectomy	3 (1.4)
Thyroid Lobectomy	2 (0.9)
**Complications**	
Yes	39 (17.6)
No	182 (82.4)

**Figure 1:**
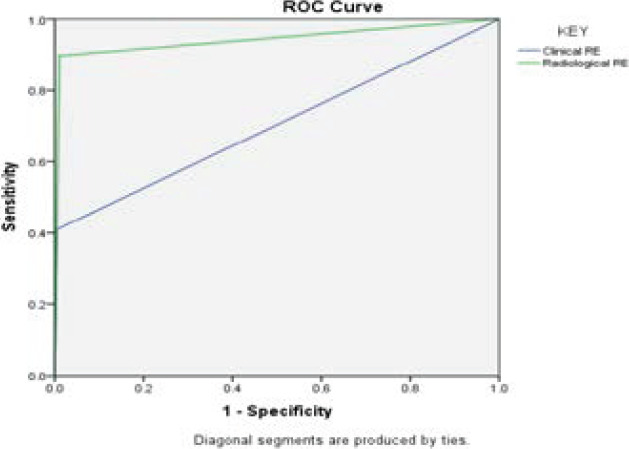
ROC curve for clinical RSG and radiological RSG

The presence of RSG on thoracic inlet radiograph accurately predicted intraoperative finding of retrosternal extension in about 94.6% of the case with an area under ROC curve of 0.943 as shown in figure 1.

Complications were recorded in 39 (17.6%) of the patients. Of these 39 patients, hypocalcaemia with mild symptoms was the most common complication and was seen in 38.5% of them. Other significant complications were seroma 15.4%, unilateral laryngeal nerve injury, non-compressive haematoma and severe hypocalcaemia account for 7.7% each. Other rare complications are as shown in Table 3.

**Table 3:** The proportion of observed complications of thyroidectomy

Types of Complications (n=39)	Frequency	Percentage (%)
Hypocalcaemia	15	38.48
Seroma	6	15.38
Non-compressive haematoma	3	7.69
Unilateral recurrent laryngeal nerve injury	3	7.69
Severe hypocalcaemia	3	7.69
Wound Infection	2	5.13
Thyroid storm	2	5.13
Anaphylactic reaction (anaesthetic agent)	1	2.56
Bilateral recurrent laryngeal nerve injury	1	2.56
Tension haematoma	2	5.13
Wound dehiscence	1	2.56

## Discussion

The occurrence of retrosternal goitre varies directly with the prevalence of cervical goitre; thus, it is more common in areas of endemic goitre. 12,13 The frequency of retrosternal goitre in this review was 17.6% and it was defined as any extension of the thyroid gland beyond the thoracic inlet intraoperatively. The reported rate of retrosternal goitre varies from less than 1% to 26.4%. 5,6,14

This depends on the region the study was conducted and the definition of retrosternal goitre in a particular study. The purpose of imaging on clinical suspicion of RSG is to establish diagnosis and evaluate the need for extra-cervical surgical access. 15,16 In this review, thoracic inlet plain radiograph was routinely done for patients with WHO grade II and III goitre and it established the diagnosis of retrosternal goitre in 16.7% of the patients but it could not be used to determine the need for extra-cervical surgical access. The extra-cervical approaches include manubriotomy, median sternotomy or thoracotomy and have been reported to be necessary in up to 3.1%, 6.6% and 4% of patients with RSG respectively. 11 In this study 5.4% of those with retrosternal goitre required median sternotomy for adequate surgical access. This is similar to the findings in other studies which showed that most retrosternal goitre can be safely removed through a cervical surgical access and that median sternotomy or thoracotomy for a secondary retrosternal goitre is an exception. 13,16,17-19 The indications for median sternotomy or thoracotomy include extension of a goitre below the aortic arch, large thyroid tissue extending towards tracheal bifurcation, and ectopic thyroid tissue in the mediastinum (primary RSG). 20 None of the patients in this study had median sternotomy. Thoracic inlet view radiograph accurately diagnosed retrosternal goitre in 37 (94.8%) of the patient who presented with RSG in the series with a sensitivity of 94.6% and specificity of 97.8%, however it could not be used to determine the need for extra-cervical surgical access. CT scan and magnetic resonance imaging which could clearly depict the lower margin of a RSG and its attachments to adjoining structures are preferred in planning for surgery especially where a thoracic surgeon may not be easily reached intra-operatively if the need arises. Some studies reported that CT scan could accurately diagnose retrosternal goitre in 100% of the cases and the findings correlate well with the intraoperative findings. 21 In this study 2 patients (5.4%) of those with clinical and/or thoracic inlet radiograph suspicious for RSG had CT scan. They had clinical features of superior vena cava compression and dyspnoea, and there were considerations for cervico-thoracic access on clinical grounds and CT scan was done to clarify this. The CT scan showed RSG extending to the ach of aorta. Both had cervical incision and manubriotomy and the CT scan findings correlated with the intraoperative findings. Retrosternal goitre has been classified according to the CT scan finding by some authors to allow for the prediction of the need for extra-cervical surgical access. One of the popular classifications based on a systematic review which included 2426 patients is as follows; class 1 – RSG above the aortic arch, class 2 – RSG extending from the arch of aorta to the pericardium and class 3 – RSG extending below the right atrium. There is need to prepare for extra-cervical access for class 2 and 3, as these group will likely require manubriotomy and median sternotomy respectively. 11 This is a useful classification especially in the sub-Saharan African region where many centres have no cardiothoracic surgeon and will require properly planning before operating on such categories of patients or they may be transferred to the appropriate facility.

In total, the morbidity rate was 17.6% (39 patients) but there was no mortality. Mild hypocalcaemia was the most common complication, it accounts for 38.5% and unilateral recurrent laryngeal nerve injury 7.7% of the complications. This represents 3.1% and 1.34% of the total patients respectively. These are the two common complications usually encountered after total thyroidectomy. 22 The frequency of hypocalcaemia in this study is similar to the finding of Sakkary et al 21 in Egypt but lower compared to other studies. 23,24 Severe hypocalcaemia requiring administration of intravenous calcium gluconate was recorded in 3 patients. All the case of hypocalcaemia were transient and resolved within 4 weeks post thyroidectomy.

## Conclusion

Thoracic inlet plain radiograph is a useful imaging study for screening patients with goitre for retrosternal extension, however it could not be used to determine the need for extra-cervical surgical access and planning for surgery. Therefore, it can be omitted in patients' evaluation, thereby reducing the cost of care and exposure to radiation. When there is a strong concern for possible extra-cervical surgical access based on clinical presentation, CT scan should be used in further evaluation when feasible or patient referred to where thoracic surgeon will be available to join in the surgery.
